# Interpreting the Early Cancer Peak in Immune-Mediated Inflammatory Diseases. Comment on Giordani et al. Cancer Association in Patients with Immune-Mediated Inflammatory Diseases: A Five-Year Nationwide Italian Cohort Study. *Cancers* 2026, *18*, 1027

**DOI:** 10.3390/cancers18213449

**Published:** 2026-10-26

**Authors:** Alper Gumustepe, Esra Gumustepe

**Affiliations:** 1 Department of Rheumatology, Atatürk Sanatorium Training and Research Hospital, Ankara 06290, Türkiye; 2 Department of Radiation Oncology, Ankara University Faculty of Medicine, Ankara, 06620 Türkiye

Giordani et al. reported a 32% higher overall cancer occurrence among patients with immune-mediated inflammatory diseases (IMIDs), with the strongest association during the first year after cohort entry and a progressive decline thereafter [1]. The authors suggest that this temporal pattern supports systemic inflammation, rather than treatment, as the principal driver of malignancy. Although biologically plausible, the observed pattern may reflect several distinct mechanisms and should not be interpreted as direct evidence of inflammation-driven carcinogenesis.

The relationship between cancer and autoimmunity is bidirectional. Chronic inflammation may facilitate tumorigenesis, but an occult malignancy may also trigger systemic immune activation and rheumatic manifestations before becoming clinically apparent. This phenomenon is well recognized in inflammatory myopathies, in which malignancies frequently cluster around disease onset and may represent a paraneoplastic process [2,3]. Consequently, cancers detected during early follow-up may include pre-existing or subclinical malignancies rather than incident tumors arising after IMID onset.

Enhanced diagnostic surveillance may further contribute to the early peak. Patients with IMIDs commonly undergo repeated clinical assessments, laboratory monitoring, imaging, and evaluation before and during immunosuppressive treatment. Such intensified healthcare contact increases the probability of detecting otherwise asymptomatic malignancies. This is particularly relevant because the comparison group consisted of patients hospitalized for injuries or poisoning, who may not have undergone comparable longitudinal surveillance.

The definition of cohort entry introduces additional uncertainty. Patients entered the cohort following hospitalization in 2018, whereas IMID diagnoses could have been recorded during the preceding five years. Therefore, the first year of follow-up represents the first year after index hospitalization, not necessarily the first year after IMID diagnosis or disease onset. Incident and longstanding IMIDs were consequently analyzed together, despite potentially different temporal relationships with cancer. Moreover, without longitudinal data on disease activity, inflammatory markers, or treatment exposure, the declining association cannot establish either improved inflammatory control or a protective effect of anti-inflammatory therapy.

The site-specific findings also warrant etiological distinction. Hematological malignancies may plausibly reflect chronic B-cell activation and immune dysregulation [4], whereas lung and bladder cancers are strongly associated with tobacco smoking, as well as other established environmental and occupational carcinogenic exposures [5,6]. The absence of smoking data is particularly consequential for the lung cancer association. Smoking is a major determinant of lung cancer and an established risk factor for seropositive rheumatoid arthritis. In a population-based cohort, patients with rheumatoid arthritis who had ever smoked had almost seven times the lung cancer risk of never-smoking population controls [7]. Adjustment for chronic respiratory diagnoses cannot substitute for smoking status, pack-years, or cessation history.

Sensitivity analyses excluding cancers detected within the first 12–24 months, restricting exposure to incident and well-defined autoimmune diseases, and accounting for healthcare utilization and smoking would help distinguish persistent disease-related risk from reverse causation and differential ascertainment. Overall, the findings provide important evidence of an association between IMIDs and malignancy; however, the temporal pattern may reflect occult cancer-associated autoimmunity, intensified surveillance, heterogeneous disease duration, shared exposures, and disease-specific immune mechanisms rather than a uniform carcinogenic effect of systemic inflammation.

## Data Availability

No new data were created or analyzed in this study. Data sharing is not applicable to this article.
